# Genetic and phenotypic similarity across major psychiatric disorders: a systematic review and quantitative assessment

**DOI:** 10.1038/s41398-024-02866-3

**Published:** 2024-03-30

**Authors:** Vincent-Raphael Bourque, Cécile Poulain, Catherine Proulx, Clara A. Moreau, Ridha Joober, Baudouin Forgeot d’Arc, Guillaume Huguet, Sébastien Jacquemont

**Affiliations:** 1 CHU Sainte-Justine Azrieli Research Center, Université de Montréal, Montreal, QC Canada; 2 Imaging Genetics Center, Stevens Institute for Neuroimaging and Informatics, Keck School of Medicine, University of Southern California, Marina del Rey, CA USA; 3 Douglas Mental Health University Institute, Department of Psychiatry, McGill University, Montréal, QC Canada

**Keywords:** Diagnostic markers, Schizophrenia, Autism spectrum disorders, Bipolar disorder, Depression

## Abstract

There is widespread overlap across major psychiatric disorders, and this is the case at different levels of observations, from genetic variants to brain structures and function and to symptoms. However, it remains unknown to what extent these commonalities at different levels of observation map onto each other. Here, we systematically review and compare the degree of similarity between psychiatric disorders at all available levels of observation. We searched PubMed and EMBASE between January 1, 2009 and September 8, 2022. We included original studies comparing at least four of the following five diagnostic groups: Schizophrenia, Bipolar Disorder, Major Depressive Disorder, Autism Spectrum Disorder, and Attention Deficit Hyperactivity Disorder, with measures of similarities between all disorder pairs. Data extraction and synthesis were performed by two independent researchers, following the PRISMA guidelines. As main outcome measure, we assessed the Pearson correlation measuring the degree of similarity across disorders pairs between studies and biological levels of observation. We identified 2975 studies, of which 28 were eligible for analysis, featuring similarity measures based on single-nucleotide polymorphisms, gene-based analyses, gene expression, structural and functional connectivity neuroimaging measures. The majority of correlations (88.6%) across disorders between studies, within and between levels of observation, were positive. To identify a consensus ranking of similarities between disorders, we performed a principal component analysis. Its first dimension explained 51.4% (95% CI: 43.2, 65.4) of the variance in disorder similarities across studies and levels of observation. Based on levels of genetic correlation, we estimated the probability of another psychiatric diagnosis in first-degree relatives and showed that they were systematically lower than those observed in population studies. Our findings highlight that genetic and brain factors may underlie a large proportion, but not all of the diagnostic overlaps observed in the clinic.

## Introduction

Psychiatric disorders such as schizophrenia (SCZ), bipolar disorder (BD), major depressive disorder (MDD), autism spectrum disorder (ASD), and attention deficit hyperactivity disorder (ADHD) are highly heritable, with genetic variance explaining from 34% to 77% of phenotypic variance [1]. Genome-wide association studies (GWAS) have successfully identified genetic variants accounting for part of this heritability, yet an important finding is that nearly 75% of significant genetic loci are shared by at least two disorders [2]. The term “pleiotropy” refers to this finding, i.e., the same genetic locus or variant contributes to more than one phenotype or diagnostic group [3]. Statistical methods to estimate heritability have been extended to measure genetic correlations (*r*_g_) which represent the average effect of pleiotropy across all contributing genomic variants.

A similar scenario has played out in neuroimaging across psychiatric disorders. Initial brain-wide association studies have sought to identify neuroimaging alterations linked to specific diagnostic categories. However, brain differences that are specific to a diagnostic category are yet to be identified. Instead, the field has progressed in characterizing neural substrates shared across disorders. Studies have suggested that neuropsychiatric disorders may be related to similar hubs of vulnerability. Latent dimensions across disorders have been identified at the structural [4] and functional MRI levels [5]. Similar to genetic correlation (*r*_g_), commonalities in brain structural and functional connectivity have been replicated in several studies. Several studies have queried the origins of these MRI correlations across psychiatric disorders by comparing them to correlations observed at other levels of observation, such as levels of gene transcription in the brain [6] and common genetic variants [7–9].

Quantifying the similarities between psychiatric disorders is also relevant to clinical practice. Pleiotropy manifests clinically by the familial aggregation of mental disorders that transcends diagnostic boundaries [10, 11], as well as the widespread co-occurrence of diagnoses in the same individuals or comorbidity [12]. A diagnosis of any mental disorder increases the risk of receiving a second distinct diagnosis [13, 14]. Moreover, co-occurring disorders (not the primary diagnosis) may account for 11–83% of disability observed in patients [15]. The process of differential diagnosis and triage to specialized clinics requires substantial resources delaying access to appropriate care [16], even though there is increasing evidence that many therapeutic interventions are effective across different diagnoses [17]. Especially, the efficacy of early-intervention programs is not specific to initial diagnosis [18].

Although there have been several anecdotal reports comparing the degree of overlap between psychiatric disorders at two levels of observation [6, 8, 9, 19, 20], a systematic investigation across all studies and levels of observation has not been carried out. We hypothesize that overlap at the molecular scale (i.e., genetic and transcriptomic) between disorders and traits may lead to similar overlaps at the macroscopic scale, such as large-scale functional networks. This study aims at assessing the degree of similarity between disorders at all levels of organization. To do so, we performed a systematic review of genetic and phenotypic correlations assessed across five psychiatric disorders.

## Methods

### Search strategy and selection criteria

This systematic review adhered to the PRISMA guidelines. We searched PubMed and EMBASE for studies published between January 1, 2009, and September 8, 2022. Keywords and index terms referred to “cross-disorder”, “pleiotropy”, “major psychiatric disorders”, or at least four among five prespecified diagnostic groups (full search strategy in Supplement 1). The prespecified diagnoses were SCZ, BD, MDD, ASD, and ADHD, which we selected for the availability of data since the first cross-disorder genetic study [2].

Abstracts were screened by congruent independent decision or joint discussion and consensus of two reviewers (VRB, CPoulain, CProulx). We included original observational studies, including meta-analyses, written in English, including at least four out of the five prespecified diagnostic groups, with or without controls as relevant, and presenting any measure of pairwise genetic or phenotypic similarity or overlap measures for all group pairs.

Full-text articles were screened by one reviewer (VRB), and all decisions were confirmed by a second reviewer (CPoulain, CProulx). Any conflicts were resolved with the senior author (SJ). Three exclusion criteria were added at the full-text review step: papers presenting similarity measures only valid in one direction from one diagnosis to another, similarity measures directly dependent on sample size (e.g., count of shared genome-wide significant loci), and absence of effect-size metric (only *p*-values). The systematic review process was performed using the Covidence web-based platform.

### Data analysis

Two reviewers (VRB and CPoulain or CProulx) independently extracted effect sizes for pairwise similarity measures regarding the five pre-specified diagnoses, as well as for other DSM-defined diagnoses represented in a landmark genetic study [2]. We contacted authors when the data was not accessible in the publication. One reviewer (VRB) extracted the number of cases, cohort size, and case definition.

The quality of included case-control studies was assessed using the Newcastle–Ottawa quality assessment scale for case-control studies [21] by discussion and consensus rating of two reviewers (VRB and CPoulain or CProulx).

We compared the pattern of similarity between all study pairs, with the Pearson correlation coefficient across disorder pairs as the primary outcome. This method was previously applied to compare patterns of similarity among psychiatric disorders [6, 8, 19, 20, 22]. We used complete pairwise observations for all comparisons with data available for at least five disorder pairs and adjusted for multiple comparisons with the Benjamini and Hochberg false-discovery rate method. To evaluate the impact of methodological choices of excluding some diagnostic groups, we conducted a sensitivity analysis by including all disorders that were only investigated in a subset of studies (Anorexia nervosa, Anxiety disorders, OCD, Tourette syndrome, PTSD, and Alzheimer’s disease).

We summarized the correlation matrix using principal component analysis, treating disorder pairs as variables, and considering each study as an observation of the extent of overlap between disorders. Imputation for missing data was done with R package missMDA [23] and principal component analysis with FactoMineR [24]. We presented the projections of disorder pairs on dimension 1, the proportion of variance explained, and the contributions (cos^2^) of each study. We obtained confidence intervals by bootstrap methods [25]. We evaluated the impact of having excluded some studies with partly overlapping data by recomputing the PCA while also including those studies.

We interpreted the genetic correlations by converting them into a clinically meaningful metric: the relative risk of a different psychiatric diagnosis in first-degree relatives of individuals with a “primary” diagnosis. To do so, we used the liability-threshold model of complex traits using statistical methods that have been previously published [26, 27]. The latter integrates information on (1) the lifetime prevalence of the disorders [28], (2) their heritability, twin-based [1] and family-based [10], and (3) genetic correlations between disorders. For this analysis, we used the most recent genetic correlation estimates from the PGC-CDG2 study [2]. We then compared the predicted risk to the observed risk previously reported in the literature for first-degree relatives [29–32].

To compare our results with the trends in research interests, as represented by the number of publications on a topic, we searched PubMed title or abstract fields on January 25, 2024, with keywords referring to the five disorders of interest since DSM-III (Supplement 10), and we computed the Jaccard index as a measure of overlap, for each pair of disorders and each year.

For all analyses, the significance threshold was fixed at *p* < 0.05, two-sided. The data was tabulated with Microsoft Excel version 16.54 and analyses were performed with R version 4.2.2.

## Results

**Fig. 1:**
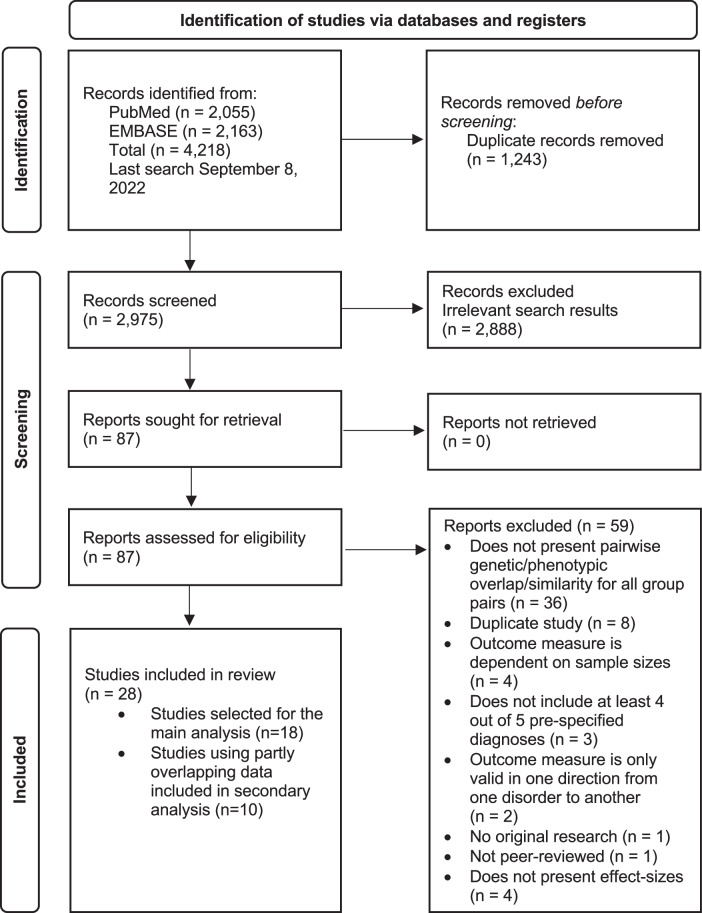
Study selection.

After removing duplicates, we screened 2975 records for eligibility (Fig. 1), and 28 studies met our inclusion criteria. Among these (Table 1), 15 studies featured genetic correlations based on single-nucleotide polymorphisms (SNPs) from case-control genome-wide studies (GWAS). Three additional studies used similar SNP-level data to infer gene-level associations and then assessed the similarity of the resulting sets of genes. Two studies computed genetic correlations based on familial aggregation of psychiatric disorders. Two studies computed gene transcription correlations between the differential gene expression profiles associated with psychiatric disorders. Five studies computed correlations across brain structural imaging measures, and one study computed correlations across resting-state functional MRI measures.

**Table 1:** Included studies and methods for computing the similarity between disorders.

Level of observation	Reference	Source of data	Method	Similarity metric	Number of cases
Single nucleotide polymorphism (SNPs)	Lee et al. [54]	PGC-CDG1	GWAS	REML, *r*_g_	32,298
	Anttila et al. [2]	Brainstorm (PGC and others)	GWAS	LDSC, *r*_g_	139,192
	Schork et al. [33]	iPSYCH	GWAS	REML, *r*_g_	49,301
	Lee et al. [55]	PGC-CDG2, 23andMe	GWAS	LDSC, *r*_g_	222,136
	Grotzinger et al. [56]	PGC, 23andMe	GWAS	LDSC, *r*_g_	437,615
	10 others^a^	PGC, 23andMe	GWAS	LDSC, *r*_g_	Supplement 2
Genes inferred from SNPs	Sey et al. [19]	PGC	Multimarker analysis of genomic annotation	Rank–rank hypergeometric test of similarity	306,405
	Li et al. [57]	PGC, 23andMe for major depression	Multimarker analysis of genomic annotation	Pearson *r*	320,606
	Gerring et al. [58]	PGC	Multimarker analysis of genomic annotation	RhoGE	344,871
Family-based genetic correlation	Wang et al. [20]	Insurance claims	Genetic effects, controlling for environment shared by siblings and within families	Familial *r*_g_	94,745
	Selzam et al. [34]	Swedish National Patient Register	Genetic effect in full siblings versus half-siblings	Familial *r*_g_	114,112
Brain transcripts (RNAs)	Gandal et al. [6]	published transcriptomics studies, PsychENCODE	brain cortex microarray of >11,229 gene transcripts	Spearman *r*	390
	Sadeghi et al. [59]	published transcriptomics studies, PsychENCODE	brain cortex microarray or RNA sequencing of 15,819 gene transcripts	Spearman *r*	1 472
Brain structural imaging (sMRI)	Kaufmann et al. [60]	ABIDE1&2, ABM, ADHD200, ADHDWUE, CIMH/CNP, HUBIN, MALTOSLO/KASP, SCHIZCONNECT1-2 and others	8 measures, machine-learning predicted brain age gap compared with chronological age based on cortical thickness, area and volume	Spearman *r*	3 427
	Opel et al. [7]	ENIGMA	34 cortical thickness and 7 subcortical measures	Pearson *r*	10,604
	Patel et al. [8]	ENIGMA	34 cortical thickness measures	Biweight midcorrelation *r*	8 641
	Radonjic et al. [22]	ENIGMA	34 cortical thickness and 7 subcortical measures	Spearman *r*	12,039
	Patel et al. [61]	ENIGMA	11 cortical surface area measures	Biweight midcorrelation *r*	8 326
Brain functional imaging, resting state functional connectivity (rsfMRI)	Moreau et al. [9]	ABIDE1, ADHD200, UK Biobank (neuroticism) for MDD, and others	Resting-state functional connectivity between 64 regions	Pearson *r*	1 022

*PGC* Psychiatric Genomics Consortium, *REML* restricted maximum likelihood, *LDSC* linkage disequilibrium score regression, *GWAS* genome-wide association study, *SNP* single-nucleotide polymorphism, *r*_g_ genetic correlation.

^a^Other studies featuring genetic correlations are provided in Supplement 2.

The median sample size among studies was 114,982.5 (total of the five diagnostic groups), minimum 390, and maximum 437,615. Quality evaluation as per the Newcastle–Ottawa scale rated studies between 4 and 7, with a median of 5 on a total of 9 points, although this scale was not designed for large meta-analytic genetic association studies. Overall, only three studies (Schork et al. [33]; Selzam et al. [34], Wang et al. [20]) achieved case representativeness and a uniform selection procedure between cases and controls.

**Fig. 2:**
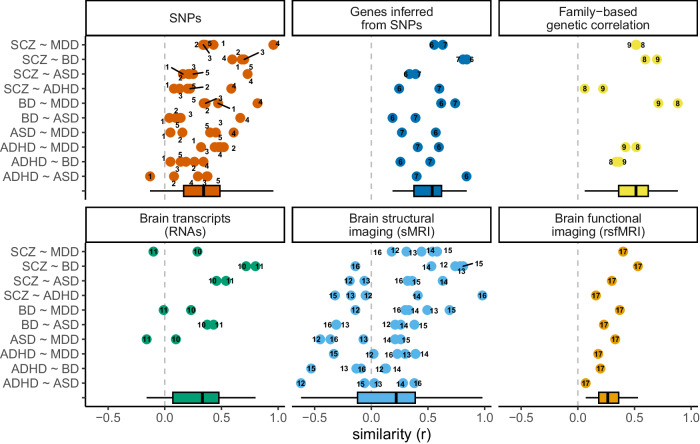
Degree of similarity between disorders, measured at different levels of observation. Each point represents the estimate of similarity (coefficient of correlation, r) between two disorders, which was measured in different studies and at different levels of observation. Descriptive statistics across studies for each level of observation are represented as boxplots; the box indicates the median, 25th, and 75th percentiles. The numbers linked to the points refer to the different studies: 1. Lee et al. [54], 2. Anttila et al. [2], 3. Lee et al. [55], 4. Schork et al. [33], 5. Grotzinger et al. [56], 6. Gerring et al. [58], 7. Li et al. [57], 8. Selzam et al. [34], 9. Wang et al. [20], 10. Gandal et al. [6], 11. Sadeghi et al. [59], 12. Kaufmann et al. [60], 13. Opel et al. [7], 14. Patel et al. [8], 15. Radonjic et al. [22], 16. Patel et al. [61], 17. Moreau et al. [5]. One study (Sey et al. [19]) that presented metrics of similarity on a scale not comparable to that of correlation coefficients was excluded from this figure.

GWAS meta-analyses have used datasets that have been updated by incorporating new cohorts year after year. Therefore, the most recent GWAS of psychiatric conditions includes all or most of the previous datasets. For statistical analyses, we selected 18 studies, thus excluding 10 studies using partly overlapping data, i.e., based on datasets that were also analyzed in more recent and larger meta-GWAS, as detailed in Table 1 and Supplement 2. The vast majority of correlation coefficients between pairs of disorders were positive (Fig. 2) throughout all 6 levels of observation.

### The pattern of similarities between disorders is consistent from the genome to transcription and to brain endophenotypes

**Fig. 3:**
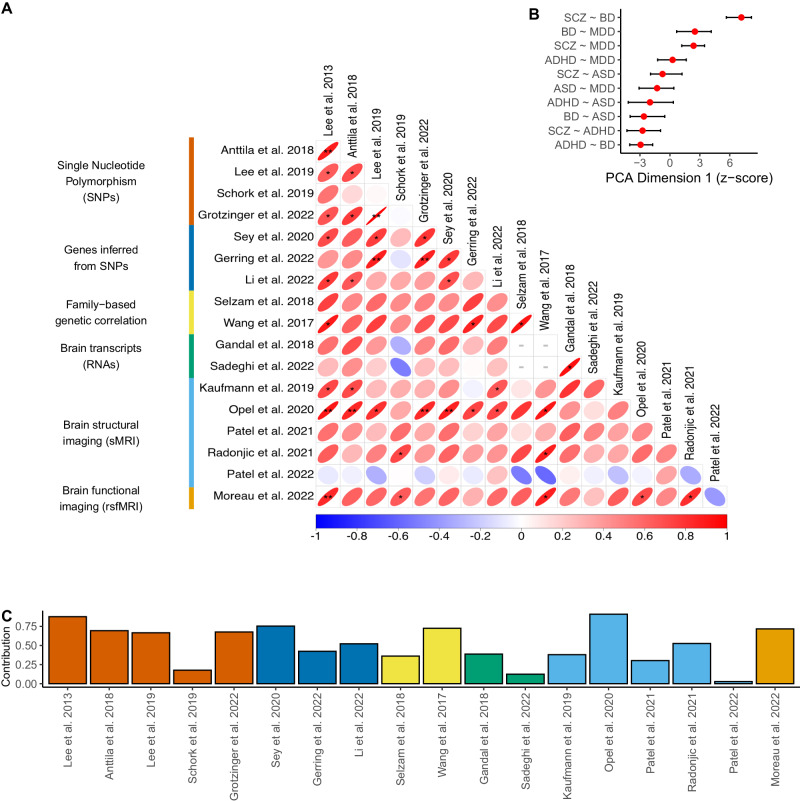
Comparisons and consensus ranking of the pattern of disorder similarities across studies. **A** Pairwise comparison matrix between 18 studies (Table 1) reporting on six levels of biological observation. For each pair of studies, we use the Pearson correlation coefficient to compare the pattern of similarities across disorder pairs. Significant correlations are marked as *unadjusted *p* < 0.05, **FDR < 0.05. Correlations with less than five pairwise disorder comparisons are marked with a gray dashed line. **B** The correlation matrix in panel A is summarized at the level of disorder pairs using principal component analysis (PCA), with a first dimension that accounts for 51.4% (95% CI: 43.2, 65.4) of variance among the 18 studies. Error bars represent 95% confidence intervals. A higher score on Dimension 1 implies that a pair of disorders has consistently higher similarity across all studies and levels of observation. **C** Contributions of individual studies to the first dimension of PCA, color-coded by the level of observation.

We tested if the pattern of similarity between disorders (SCZ, BD, MDD, ASD, and ADHD) was consistent across all 18 studies and levels of observations. We observed that 89% (132/149) of pairwise study comparisons displayed in Fig. 3 showed positive associations as measured by the Pearson correlation coefficient, of which 9 were significant after correction for multiple testing. This suggests that the pattern of similarities between disorders was conserved across different levels of observation (genetic, family, transcriptomic, and brain) as well as across studies.

We conducted sensitivity analyses to evaluate the influence of the selection of some diagnostic categories over others on our results. We included all disorders that were only investigated in a subset of studies (Anorexia nervosa, Anxiety disorders, OCD, Tourette syndrome, PTSD, and Alzheimer’s disease), and the resulting correlation matrix was 95.3% concordant (as per the concordance correlation coefficient [35]) with the one (Fig. 3A) restricted to five disorders.

To obtain a consensus ranking of similarities between pairs of disorders across all studies and levels of observation, we used principal component analysis (PCA) (Fig. 3B). The three first dimensions explained 51.4% (95% CI: 43.2, 65.4), 18.8% (95% CI: 13.5, 28.6), and 9.7% (95% CI: 6.8, 16.9) of the variance, respectively. We thus interpreted the first dimension as representing the consensus of similarities across conditions and all other dimensions representing noise. The pairs of disorders ranking the highest on the first dimension (Fig. 3B) were those with the highest level of similarity: SCZ-BD, followed by SCZ-MDD and BD-MDD.

We asked if the levels of overlap between disorders increased (convergence) or decreased (divergence) from genes to transcription to brain endophenotypes. All levels of observation showed similar loadings on dimension one (Fig. 3C). In other words, we observed a pattern of similarities between disorders which was conserved at all levels of observation.

We performed a sensitivity analysis by including all studies (i.e., those using partly overlapping data) reporting genetic correlations studies in the PCA. This did not significantly change the ranking obtained by PCA (Supplement 8).

### Clinical relevance

**Fig. 4:**
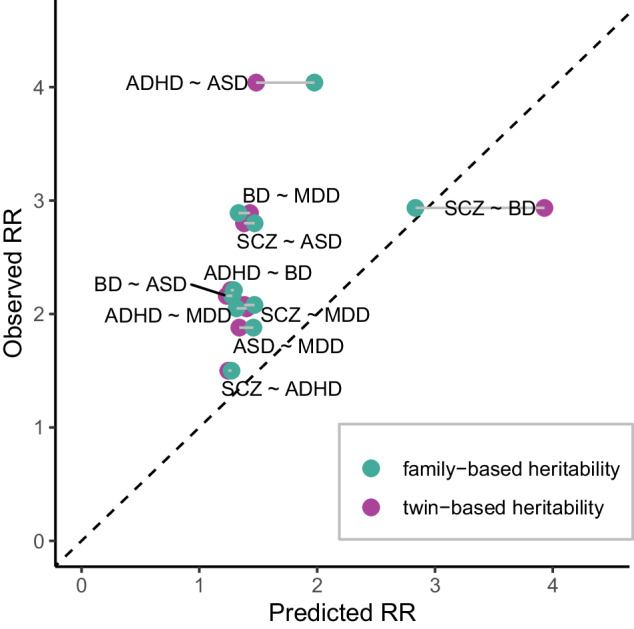
Risks in first-degree relatives across disorders. Probability in first-degree relatives across disorders displayed as relative risk (RR) compared to the lifetime risk present in the general population. Genetically predicted risks were computed using published methods and in two independent analyses, using either family-based or twin-based heritability, both of which are compared here with observed risk estimates from published population-based studies. The risks observed clinically were systematically higher than those predicted on the basis of the genetic overlap. Note that the risk ratios are valid in both directions from one disorder to another (RR_diagnosis1|diagnosis2_ = RR_diagnosis2|diagnosis1_).

To assess the clinical relevance of this biological overlap, we estimated the probability of other psychiatric diagnoses in first-degree relatives using a previously published method that uses the degree of genetic correlation as input. We compared our estimates with previously published observations (Fig. 4) [29–32]. While some estimates were concordant with previously published clinical observations overall, clinically observed risks in relatives were systematically higher than those predicted on the basis of the genetic correlation between disorders.

### Research trends on cross-disorder versus single-disorder studies

**Fig. 5:**
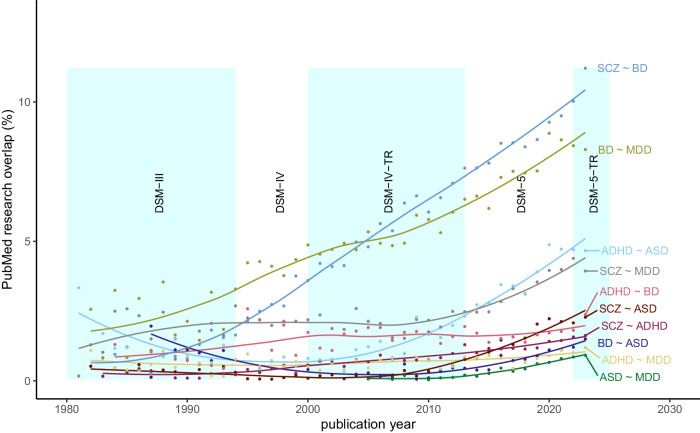
Cross-disorder research trends. Proportion of studies referenced on PubMed that address two disorders at a time, for each pair of disorders, as compared to the total number of studies for the same pair of disorders, as a function of publication year. Locally estimated scatterplot smoothing (LOESS) regression lines indicate trends over time. Shaded background colors correspond to the Diagnostic and Statistical Manual of Mental Disorders (DSM) editions.

We investigated whether the amount of research conducted on pairs of psychiatric disorders over time was congruent with the level of similarity between the same pairs of disorders (Fig. 5). In the last 10 years, we observed an increase in the proportion of research records that investigate pairs of disorders over the total including only one condition. In 2023, the proportion of publications investigating pairs of psychiatric disorders was correlated (*r* = 0.84, *p* = 0.0022) with the overall level of overlap computed across all levels of observation. The overlaps of MDD with ADHD, SCZ, and ASD were the strongest outlier disorder pairs that were understudied relative to their biological correlation.

## Discussion

In this first systematic investigation of similarities between psychiatric disorders across different levels of observation, results show that the patterns of similarities between disorders are consistent, from molecular to higher levels of organization (i.e., large-scale brain networks). This pattern can be summarized by one principal dimension, which accounts for more than half of the variance across all studies. These results suggest that important aspects of pathophysiology are shared across disorders and are not circumscribed under the boundaries of the current diagnostic classification. Interestingly, the rates of psychiatric diagnoses in relatives reported by clinical studies were systematically higher than what is predicted by genetic correlation.

The inquiry into whether the observed consistent biological similarity across disorders is the substrate of shared symptoms remains an open and pivotal question. Our systematic review, while comprehensive across biological levels, did not retrieve studies on symptom profiles or neurocognitive profile correlations across pairs of psychiatric disorders that would correspond to inclusion criteria. Nevertheless, evidence supporting the genetic underpinnings of shared symptoms emerges from focused studies on specific traits within disorder pairs. Notably, common variants associated with schizophrenia were linked to psychotic symptoms in individuals with bipolar disorder, and conversely, variants linked to bipolar disorder were associated with manic symptoms in individuals with schizophrenia [36]. Another study found that both schizophrenia and ASD-associated common variants impacted social communication in the general population, with differing effects in childhood and adolescence [37]. These shared symptom profiles may contribute to the observed high rates of co-occurrence between disorders, evident both concurrently [38] and sequentially [13, 14], as observed in community-based samples.

The finding of higher rates of occurrences of disorders in relatives [29–32] than anticipated based on genetic correlations suggests that the latter, computed solely on common variants, might be underestimated. In fact, genetic correlations are so far restricted to common variants, although pleiotropy has repeatedly been reported for rare variants with respect to neurodevelopmental and psychiatric conditions [39]. Whether rare variants show higher levels of pleiotropy and/or genetic correlation than common variants remains unknown. A recent study suggests that function-based genetic correlations computed for recurrent copy-number variants may be quite different from those observed for common variants [40].

Various factors, extending beyond genetic correlations, may contribute to the observed covariance among psychiatric disorders. Environmental exposures, such as adverse childhood experiences encompassing physical, sexual, or emotional abuse, exert cross-disorder effects [41] and might be causal [42]. However, other cross-disorder associations were also found in systematic phenome-wide investigations of genetic liability [43], and it is crucial to acknowledge that the association between environmental exposures and psychiatric disorders can also result from confounding variables or from reverse causation, as indicated by insights from mendelian randomization analyses [44]. In that regard, several other factors underline an intricate interplay between genetic and environmental factors in the etiology of psychiatric disorders. Frequent “assortative mating” of individuals with psychiatric conditions [45], within-family environmental transmission (“genetic nurture”), and multi-generational processes of social stratification (“dynastic effects”) [46] can contribute to the shaping of environments that correlate with various genetic variants and predispose individuals to psychiatric conditions.

In evaluating the extent of similarity between disorders, it becomes apparent that brain structures exhibit a more variable degree compared to other biological levels (refer to Fig. 2). This discrepancy suggests a critical reevaluation of a historical focal point, brain structures. Alternative levels of observation, such as brain transcripts (RNAs) or functional brain imaging (resting-state fMRI), emerge as possibly more aligned with the shared genetic underpinnings among psychiatric conditions. This could also be interpreted in light of previous research, which noted that brain imaging profiles linked to distinct genetic psychiatric risk variants displayed only mild correlations, and yet that these same profiles were found to be associated with highly correlated phenotypic patterns [47, 48].

A controversy concerning cross-disorder similarity is that research samples may be enriched or depleted in patients with co-occurring disorders (based on inclusion and exclusion criteria and sampling procedure) and that this biased sampling could result in over- or underestimating the levels of similarity [3]. Also, the sampling procedure of control participants can bias the estimate of genetic correlations by favoring the recruitment of “super-normal” controls [49], that is, individuals who, in addition to not having the diagnosis of interest, do not have a set of frequently co-occurring and genetically-related conditions. Such biases in the recruitment of cases and controls pose a potential challenge to the generalizability and the applicability of findings in clinical settings. It is noteworthy, however, that our systematic review also retrieved studies employing representative sampling strategies. These studies, conducted in both the general population (Schork et al. [33]; Selzam et al. [34]) and within a healthcare system (Wang et al. [20]), revealed patterns of overlap across disorders that align with findings from other studies.

### Limitations

It is of significance that this study demonstrates consistent levels of correlations both within and between levels of observation, even though study samples were recruited in different settings (clinical or research-based), different countries, and based on criteria from different DSM and ICD editions. Such heterogeneity has been previously associated with variable prevalence of these conditions, as well as variable case-control differences [50]. Despite the potential for such heterogeneity to contribute to noise or non-systematic bias when specifically assessing the concordance between levels of observation, our findings indicate a consistent covariance across levels of observation as measured by different studies. This consistency suggests that the observed pattern of covariance between psychiatric disorders remains robust, even when confronted with variations in diagnostic and inclusion criteria.

Insights from the present study could guide clinical practice and health services organizations in prioritizing joint interventions for disorders that are the most similar. The observed high degree of overlap among schizophrenia, bipolar disorder, and major depressive disorder aligns with current trends in restructuring patient care based on transdiagnostic dimensions (e.g., age of onset, episode recurrence, premorbid functioning) complementarily to the primary diagnosis [51]. Moreover, the identification of a lower but nevertheless prevalent overlap of these disorders with earlier-onset neurodevelopmental conditions (autism and ADHD) suggests the need for a comprehensive, lifespan approach to mental health. This recognition becomes particularly crucial in early-intervention settings, where the initial clinical presentations of psychiatric disorders do not fit well under the traditional diagnostic categories [18]. The broad level of overlap across psychiatric disorders questions the way resources are spent on evaluating differential diagnoses, as opposed to setting the emphasis on assessing transdiagnostic dimensions. While cross-disorder research is expanding, buoyed by initiatives such as RDoC [52] or HiTOP [53], we show that still only a minority of studies address more than one disorder.

### Supplementary information

Supplement

PRISMA checklist
